# Predaceous and Phytophagous Pentatomidae Insects Exhibit Contrasting Susceptibilities to Imidacloprid

**DOI:** 10.3390/ijms26020690

**Published:** 2025-01-15

**Authors:** Hongmei Cheng, Zhen Wang, Xiaoyu Yan, Changjin Lin, Yu Chen, Le Ma, Luyao Fu, Xiaolin Dong, Chenxi Liu

**Affiliations:** 1 Sino-American Biological Control Laboratory, Institute of Plant Protection, Chinese Academy of Agricultural Sciences, Beijing 100193, China; chenghongmei1010@163.com (H.C.); wangzhen19810@163.com (Z.W.); linchangjin163@163.com (C.L.); fuluyao5989@163.com (L.F.); 2 Department of Entomology, Yangtze University, Jingzhou 434023, China; yanxiaoyu0316@163.com (X.Y.); chenyu2632@163.com (Y.C.); male0402@163.com (L.M.); dongxl@yangtzeu.edu.cn (X.D.)

**Keywords:** imidacloprid, pentatomidae, pesticide susceptibility, toxicity

## Abstract

Imidacloprid, a widely used neonicotinoid insecticide, targets insect pests but also affects natural enemies. However, the effects of neonicotinoid insecticides on closely related insects remain unclear. We evaluated the harmful effects of imidacloprid on the phytophagous *Halyomorpha halys* and predaceous *Arma chinensis*. Bioassays revealed that imidacloprid was more toxic to *H. halys* than to *A. chinensis* and more harmful to the males than to the females of the two insects. *A. chinensis* adults recovered from imidacloprid-induced knockdown, as evidenced by restored respiratory rates, metabolic rates, and locomotion. Surviving *A. chinensis* showed reduced fecundity, suggesting a trade-off between detoxification and reproduction. Bioinformatics analysis of nicotinic acetylcholine receptors (nAChRs) and molecular docking simulations indicated a lower diversity of the nAChR gene family in *A. chinensis* than in *H. halys*, with weaker binding to imidacloprid, consistent with the relatively low toxicity of the insecticide in this species. This might account for the susceptibility differences to imidacloprid between the species. These findings underscore the efficacy of imidacloprid against *H. halys* and provide insights into the toxicities of neonicotinoids to target and non-target insects.

## 1. Introduction

Insect pest management relies heavily on pesticides and natural enemies [1,2,3]. Owing to the prevalent use of pesticides [4], their effect on non-target organisms is a significant concern [5,6]. Chemical insecticides often induce non-lethal physiological, developmental, and behavioral effects; they also affect insect longevity and fecundity [7,8]. Therefore, the selective toxicity of insecticides toward non-target organisms is essential [9].

Imidacloprid is a neonicotinoid insecticide widely used to control several insect pests [10], being particularly effective against sucking pests and various coleopteran, dipteran, and lepidopteran pest species via foliar, soil, and seed application [11,12,13]. The use of neonicotinoids has important negative effects on non-target organisms [14,15]. Imidacloprid exhibits selective toxicity in insects [16] by strongly binding to nicotinic acetylcholine receptors (AChRs) [17,18]. These are crucial excitatory receptors in the insect central nervous system [19,20] that are expressed by a diverse gene family [21,22]. Despite the existing knowledge of their heteromeric nature [23], the precise subunit composition of native nAChRs in insects remains unknown.

Pentatomidae, within the suborder Heteroptera [24], comprises a wide range of invasive agricultural pests and natural enemies with great economic importance [25,26,27]. Among Pentatomidae, the phytophagous *Halyomorpha halys* and predatory *Arma chinensis* are closely related species with opposite feeding habits, as confirmed by genomic [28,29,30] and morphological classifications [31,32]. *H. halys* is a globally invasive agricultural pest that damages myriad crops [28,33]. Conversely, *A. chinensis* individuals show promise as biological control agents against lepidopteran and coleopteran pests [34].

Typically, closely related species with similar morphological features exhibit similar susceptibilities to insecticides [35,36]. However, recent studies have not addressed the effects of neonicotinoid insecticides on closely related insects, particularly when one is harmful and the other beneficial. Incidentally, *H. halys* and *A. chinensis* are inevitably exposed to pesticides simultaneously; therefore, studying the toxicity of imidacloprid to non-target *A. chinensis* is crucial.

We assessed the toxicity of imidacloprid to these two insects under laboratory conditions and evaluated the respiratory, metabolic, locomotor, and reproductive performance of *A. chinensis*. Additionally, we explored the potential mechanism of imidacloprid toxicity through genome-wide analysis and molecular docking approaches. To our knowledge, this is the first report on the toxicity of imidacloprid toward two morphologically related insects with opposite feeding habits.

## 2. Results

### 2.1. Acute Toxicity of Imidacloprid and Predatory Insect A. chinensis Recovery

The KD_50_ values of imidacloprid were higher in *A. chinensis* than in *H. halys*. Specifically, the 24 h KD_50_ for males and females was 1048.682 mg/L and 1210.253 mg/L, respectively, in *A. chinensis* but 339.568 mg/L and 433.447 mg/L, respectively, in *H. halys*. The result showed that *A. chinensis* had a higher tolerance to imidacloprid than *H. halys* and that females had a higher tolerance than males in both species.

**Figure 1:**
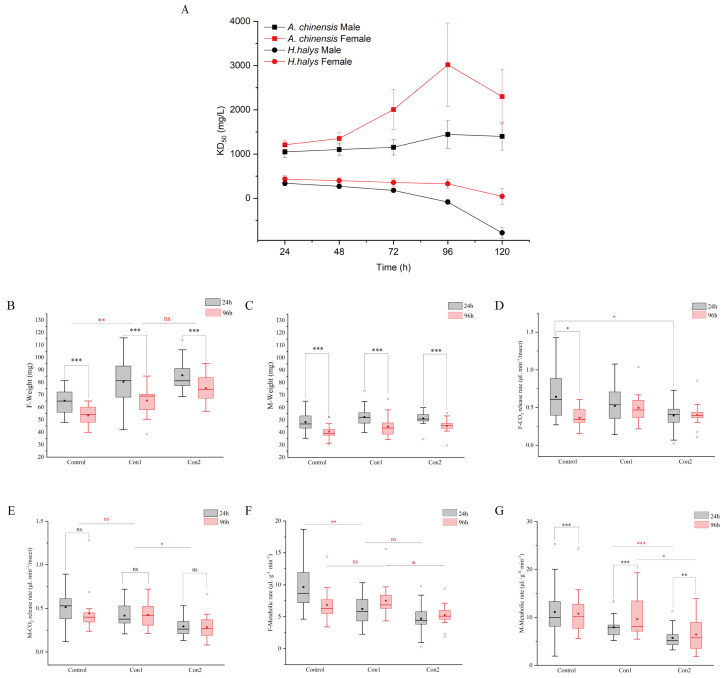
(**A**) The toxic effects of imidacloprid on *Arma chinensis* and *Halyomorpha halys* 24, 48, 72, 96, and 120 h post exposure. The points indicate the KD_50_ value (n = 3, error bars, SEM) used to assess changes, determined using a probit regression model. Analysis of the weight, respiration, and metabolism of *A*. *chinensis* exposed to different concentrations of imidacloprid [Control, Con1 (700 mg/L), Con2 (1900 mg/L)] for 24 h (gray) and 96 h (red). (**B**,**C**) Weight differences between male (M) and female (F) adults. (**D**,**E**) Respiratory rates. (**F**,**G**) Metabolic rates. Box plots show the median (line), mean (dotted line), 25th and 75th percentiles, and outliers (circles). * *p* < 0.05, ** *p* < 0.01, *** *p* < 0.001 and ns, not significant, for comparisons of males and females, as determined using two-way repeated measures ANOVA, followed by pairwise multiple comparisons, Bonferroni test. Black asterisks indicate intra-group comparison; red asterisks indicate inter-group comparisons. The metabolic data of the Control (96 h), Con1 (700 mg/L) (96 h), and Con2 (1900 mg/L) (24 h) males were transformed by SQRT (square root transformed).

The trend line of KD_50_ showed that both *A. chinensis* and *H. halys* were knocked down by imidacloprid within 24 h, but remarkably, between 24 and 96 h, the KD_50_ of *A. chinensis* increased while that of *H. halys* decreased, implying that *A. chinensis* recovered from knockdown, whereas *H. halys* did not (Figure 1A). The KD_50_ at 96 h was 0.38- and 1.49-fold higher than that at 24 h for male and female *A. chinensis*, respectively.

### 2.2. Respiratory Metabolism After Imidacloprid Administration

We observed no significant interaction between treatment and time in relation to the weights of *A. chinensis* (Female: F (2, 32) = 1.670, *p* = 0.204; Male: F (2, 32) = 3.240, *p* = 0.052). Furthermore, the weights of female *A. chinensis* treated with Con1 and Con2 were significantly higher than those of the control at 96 h. These results indicated that body fat consumption was slower in *A. chinensis* treated with high insecticide concentrations (Figure 1B). Imidacloprid treatment had no significant effect on the weight of male *A. chinensis* (F (2, 32) = 1.769, *p* = 0.187; Figure 1C). Time significantly affected weight loss (Female: F (1, 16) = 152.403, *p* < 0.001; Male: F (1, 16) = 362.678, *p* < 0.001).

The CO_2_ release rate in females showed a significant interaction effect between treatment and time (Female: F (2, 32) = 6.588, *p* = 0.004) but not in males (Male: F (1.443, 23.082) = 0.319, *p* = 0.659). In females, the CO_2_ release rate varied with dose at 24 h but was independent of dose at 96 h (Figure 1D). In males, imidacloprid treatment resulted in a concentration-dependent decrease in CO_2_ release rate, with time showing no significant difference on metabolic activity (F (1, 16) = 0.624, *p* = 0.441; Figure 1E).

The metabolic rate of *A. chinensis* also exhibited significant interaction effects between treatment and time (Female: F (1.551, 24.821) =5.676, *p* = 0.014. Male: F (2, 32) = 35.390, *p* < 0.001). Unlike in the control group, the metabolic rate of female *A. chinensis* treated with imidacloprid increased at 96 h (Figure 1F). In males, the metabolic rate increased only in the Con2 group (Figure 1G). Our results indicate that *A. chinensis* recovered its physiological activity after exposure to the recommended field dose (Con1) for 96 h. At this dose, the recovery ability of *A. chinensis* was independent of sex. However, at higher concentrations, the respiratory metabolic rate of female *A. chinensis* returned to normal after 96 h, while that of male *A. chinensis* did not. Meanwhile, the results showed imidacloprid is potentially safe for the predatory *A. chinensis* after exposure to the recommended field dose when employed as a biological control agent.

### 2.3. Locomotion Recovery After Imidacloprid Treatment

**Figure 2:**
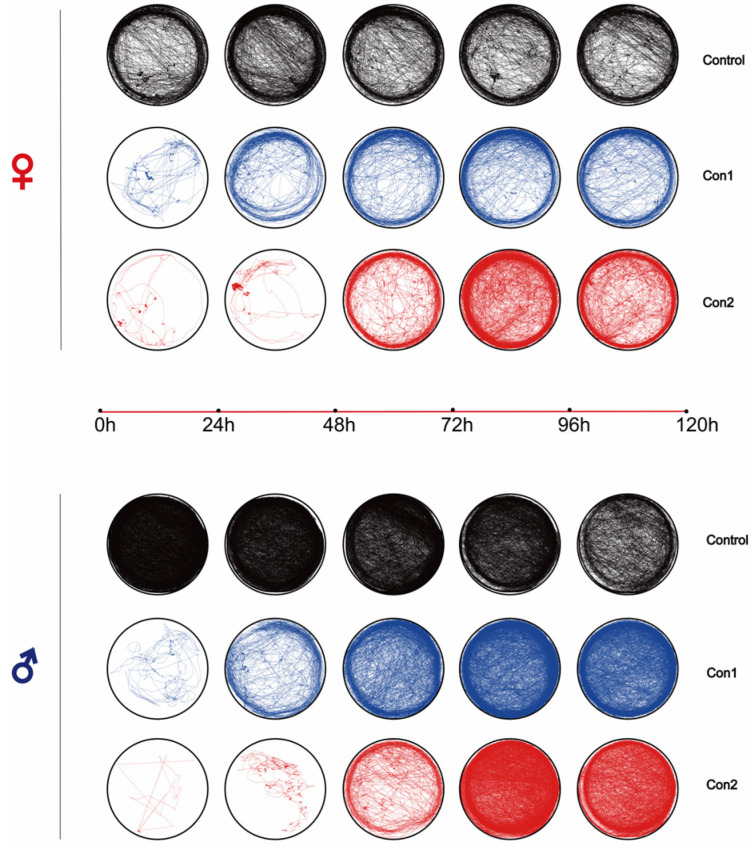
The behavioral tracking analysis of *Arma chinensis* following imidacloprid treatment. The representative trajectory of imidacloprid-treated females and males after 120 h of exposure (Control, Con1, Con2), recorded using a Luowice Y10 camera fitted with infrared lights.

**Figure 3:**
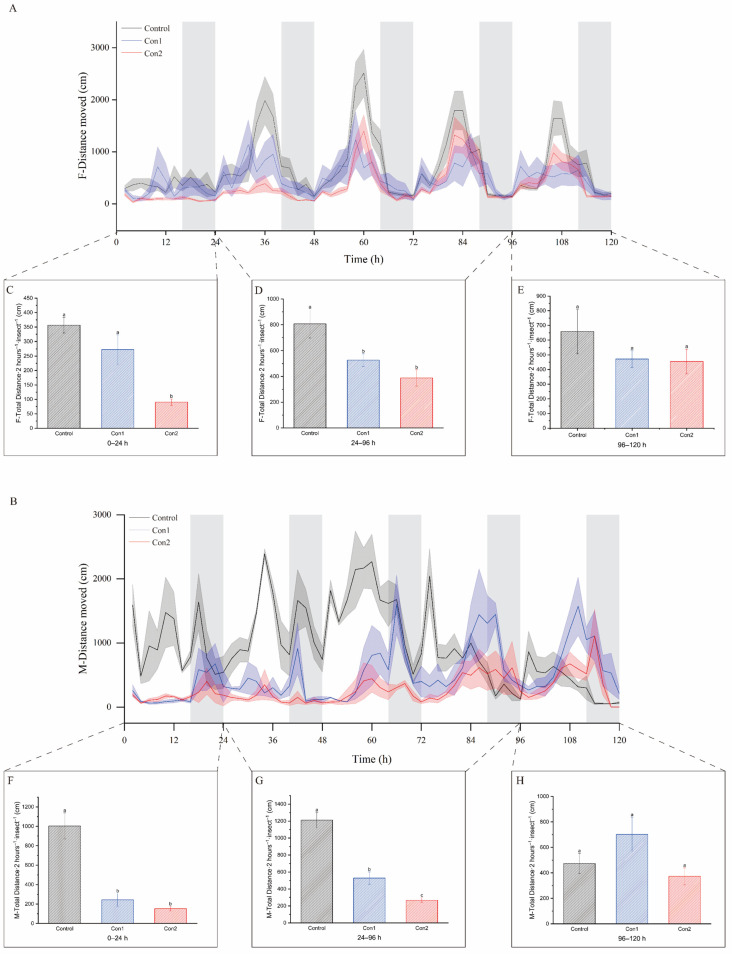
(**A**,**B**) The average distance traveled females and males after 120 h of exposure to various concentrations (control (black), Con1 (blue), Con2 (red)) of imidacloprid. The data are expressed as the means ± SEM, analyzed using one-way ANOVA and the Waller–Duncan test. Significant differences in average total distance traveled for 0–24 h (**C**,**F**), 24–96 h (**D**,**G**), and 96–120 h. (**E**,**H**). n = 15 for all groups. Means with different letters differ significantly, *p* < 0.05.

Imidacloprid significantly affected locomotor activity in a dose-dependent manner (Figure 2 and Figure 3). We calculated the total distances traveled by females and males following imidacloprid exposure (Figure 3A,B). At 24 h, *A. chinensis* movements were reduced at both concentrations of insecticides. The total distance traveled by *A. chinensis* decreased with the increase in dose from 0 to 96 h (*p* < 0.05; Figure 3C,D,F,G). However, the locomotor activity of *A. chinensis* did not differ significantly across treatments from 96 to 120 h (Figure 3E,H).

### 2.4. Reproductive Performance of A. chinensis After Recovery

**Figure 4:**
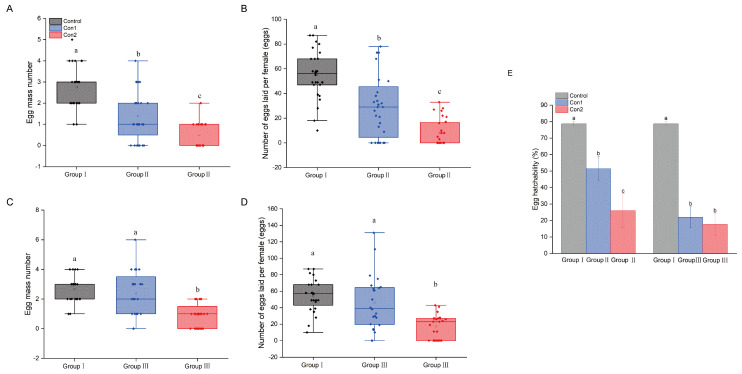
(**A**,**B**) Egg mass and number of Groups Ⅰ and Ⅱ. (**C**,**D**) Egg mass and number of Groups Ⅰ and Ⅲ. (**E**) Egg hatching rate of Groups Ⅰ, Ⅱ, and Ⅲ. Means with different letters are significantly different; Waller–Duncan test, *p* < 0.05.

Imidacloprid significantly affected the reproductive capacity of surviving adults. The number of egg clusters and eggs decreased significantly following imidacloprid treatment (Group Ⅱ) (*p* < 0.05) (Figure 4A,B). Con1 did not significantly alter the number of egg clusters and eggs per cluster, whereas Con2 significantly decreased these parameters (Group Ⅲ) (*p* < 0.05) (Figure 4C,D). Furthermore, the egg hatching rates after treatment with imidacloprid were significantly lower than that after the control treatment (Figure 4E).

### 2.5. Phylogenetic and Domain Organization Analysis of nAchR in A. chinensis and H. halys

In the *A. chinensis* proteome, eight non-redundant nAchR family members were identified, whereas *H. halys* possessed 24 non-redundant nAchR family members. These genes were renamed based on annotation information (Tables S1 and S2). The unrooted phylogenetic tree results (Figure S1A) revealed that *A. chinensis* nAchRs clustered into seven groups. The AChR alpha3-like group included two AChRs.

**Figure 5:**
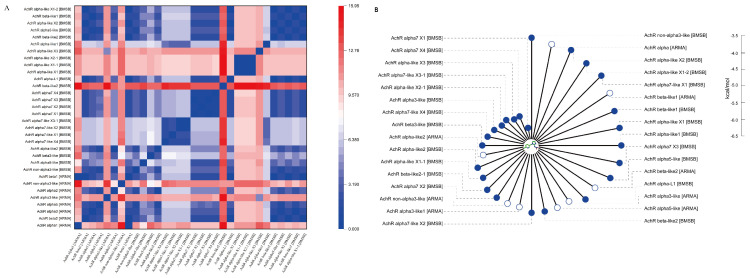
(**A**) The root mean square deviations of the nAchR subunit from *Arma chinensis* and *Halyomorpha halys*. (**B**) The minimum binding energy of imidacloprid with the nAchR subunit of *A. chinensis* (open circles) and *H. halys* (solid circles). The distance from the point to the center represents the binding energy values; the longer the distance, the weaker the binding capacity.

Homology analysis demonstrated high similarity among nAchR protein subunits; however, in *A. chinensis* the number nAchR family members was significantly contracted compared with that of *H. halys* (Figure S1B,C), suggesting an evolution-driven adaptive response in *A. chinensis* [37]. RMSD revealed five similar nAchR protein subunits between *A. chinensis* and *H. halys* (RMSD ≤ 2; Figure 5A).

### 2.6. Binding Ability of Imidacloprid to Acetylcholine Receptors of A. chinensis and H. halys

Molecular docking showed that the binding ability of the nAchR subunit in *A. chinensis* was weaker than that in *H. halys* (Figure 5B). Specifically, the lowest binding energy observed for *A. chinensis* nAChR bound to imidacloprid was −5.4 kcal/mol, whereas it was −6.1 kcal/mol for *H. halys* nAChR. Molecular docking simulations of the nAChR subunits in *A. chinensis* (Figure S2A) and *H. halys* (Figure S2B) revealed the stronger binding of imidacloprid and acetylcholine to the *H. halys* receptor than to the *A. chinensis* receptor. Therefore, the enhanced insecticide resistance observed in *A. chinensis* may be attributed to the weaker binding affinity of its nAChR to imidacloprid.

## 3. Discussion

In this study, *A. chinensis* recovered from KD, whereas *H. halys* did not. Our results underscore the importance of considering recovery dynamics in assessing the effects of pesticides on non-target insects, especially beneficial insects. That *H. halys* individuals did not recover after treatment with imidacloprid aligns with previous studies [38,39]. Females generally exhibited higher tolerance to imidacloprid than males in both species, in contrast to previous results showing lower sensitivity of male *H. halys* to nicotinoid insecticides [40].

Our observations suggest that imidacloprid significantly reduced *A. chinensis* locomotor activity in a time- and dose-dependent manner, implicating potential mechanisms by which the insect develops resistance to insecticides through reduced movement, respiration, and metabolic rates. In contrast to previous studies [41,42], the locomotor abilities and metabolism of *A. chinensis* were restored from KD over time. Although all studies were conducted under fasting conditions, our observations highlight the ability of *A. chinensis* to recover from imidacloprid-induced KD.

A previous study reported detoxification and neurotransmitter clearance drove the recovery of *A. chinensis* from β-cypermethrin-triggered knockdown [43], indicating the predatory bug had higher resilience to the insecticide. This research likewise found that the recovery of *A. chinensis* from knockdown following the application of imidacloprid. The changes in the profile of *A. chinensis* respiration through the entire recovery process showed that the predator was coping with the stress of imidacloprid and experiencing the metabolic effects of the insecticide in vivo. In contrast, quantitative data on locomotion during the 120 h experimental period can accurately and directly reflect the state of the insect.

Disruptions caused by pesticide exposure can interfere with normal reproductive processes [44]. Our study indicated that despite imidacloprid exposure, *A. chinensis* retained the ability to lay eggs and reproduce; however, the number of egg clusters and eggs laid was significantly lower than that in the control group. Maternal exposure to insecticides can decrease fecundity and impair offspring development [9,45,46]. Moreover, our results suggest that *A. chinensis* exhibits a typical trade-off between detoxification and reproduction, which are two highly energy-demanding processes [47]. Imidacloprid-exposed couples were not evaluated in this study owing to mortality during mating and spawning post treatment. This underscores the significant effect of imidacloprid on the mating and reproduction of non-target *A. chinensis.*

Protein domains are evolutionally conserved in primary and tertiary structures [48,49]. Considering the limited information on the crystal structure of the nAchR in *A. chinensis* and *H. halys*, we used the conserved nature of protein domains to provide insights into its imidacloprid-binding mechanism. Bioinformatics indicated significant differences in the number of nAChRs and RMSD values between *A. chinensis* and *H. halys* but structural similarities in more than half of the proteins. The molecular docking results aligned with the toxicological tests, affirming the stronger binding affinity of *H. halys* with imidacloprid. Therefore, we hypothesize that the contraction of the nAchR family in *A. chinensis* is a contributing factor to its heightened sensitivity to imidacloprid.

Although this study provides preliminary results on insecticide resistance in non-target insects, there are still limitations to our study. For example, the barrier characteristics of the insect cuticle, and the specific transport and detoxification mechanisms involved in insecticide sequestration and elimination, can contribute to insecticide tolerance [50]. Future studies should take these factors into consideration in order to better understand the mechanisms of insecticide tolerance.

## 4. Materials and Methods

### 4.1. Insect Cultivation

*A. chinensis* and *H. halys* individuals were collected from a laboratory population that had been maintained for more than 35 generations. *A. chinensis* individuals were maintained in a rearing cage made from a plastic bottle (15.0 × 15.0 cm) and fed *Antheraea pernyi* pupae. *H. halys* individuals were fed corn while housed in boxes (34.5 × 23.3 × 16.0 cm).

The insects were reared under controlled conditions of 26 ± 1 °C, 65 ± 5% relative humidity, and a 16 h light/8 h dark photoperiod. The populations were not exposed to insecticides during laboratory rearing. Adults aged 3–5 days post emergence were individually housed in 10 × 15 cm plastic bottles (one insect/bottle) for subsequent experiments.

### 4.2. Toxicity Bioassays

Imidacloprid (25%) was obtained from the market (Hebei, China), dissolved in distilled water, and prepared as a series of concentration gradients following the manufacturer’s recommended field doses. Distilled water was used as the parallel solvent control. Toxicity was assessed using a modified topical application method [51]. The insects were placed into 10 × 15 cm plastic bottles 3–5 days after emergence, and 5 μL of diluted imidacloprid or water (control) was applied to the pronotum region of a single adult, which was then transferred to a transparent bottle covered with gauze and secured using a rubber band. Each treatment included three replicates per insecticide. Overall, 420 adults of *A. chinensis* and *H. halys* (210 males and 210 females of each species) received insecticide treatment.

The endpoint assessed in the insect subject bioassay was “knockdown” (KD). Resistance to imidacloprid was assessed by determining the concentration required to knock down half of the insects (KD_50_) [52]. KD effects were recorded every 24 h over a 5-day period, with insects considered knocked down if they showed minimal or no response to brush probing.

### 4.3. Respiratory Metabolism Rate

Respiratory rate bioassays were performed 24 and 96 h post exposure to two doses of imidacloprid (recommended field dose: Con1, 700 mg/L; exceeding maximum recommended field dose: Con2, 1900 mg/L) and distilled water as a control.

The CO_2_ release rate was monitored in adults that survived KD after exposure to Con1, Con2, and the control. CO_2_ production (μL·min^−1^·insect^−1^) was measured using a TR3C CO_2_ analyzer (Sable System International, Las Vegas, NV, USA). Adult *A. chinensis* individuals were placed in respirometry chambers (25 mL) connected to a closed system. After acclimatization, CO_2_ production was continuously measured for 7 min using an infrared reader inside the chamber. Weights were recorded before and after the experiment using an analytical scale (CPA124S, Sartorius, Germany). Seventeen replicates were used per concentration of each insecticide and in the control in a completely randomized design.

### 4.4. Locomotion Tracking

Following imidacloprid or water treatment, 90 *A. chinensis* adults (45 males and 45 females) were randomly selected and placed individually in Petri dishes (9 cm diameter). Insect behavior was tracked and recorded at a resolution of 720 × 480 pixels at 10 frames/s using a Luowice Y10 video camera (Shenzhen Qixin Electronics Co. Limited, Shenzhen, China) equipped with infrared lights. All areas with test predators were numbered to allow the recording and analysis of the behavior of individual insects during the 120 h experimental period. The camera output was fed into a standard computer with Etho Vision XT14 (version 14.0, Noldus, Wageningen, The Netherlands), which was used to create videos for the posterior analysis of locomotor activity. The experimental system was stored in a temperature-controlled room at 24 ± 1 °C. Subsequently, the data were analyzed using Origin (version 9.9).

### 4.5. Reproductive Performance of A. chinensis

To evaluate the reproductive performance of *A. chinensis* following insecticide exposure, three mating combinations were tested: unexposed couples (Group Ⅰ); exposed females and unexposed males (Group Ⅱ); and exposed males and unexposed females (Group Ⅲ). Twenty-eight mated couples per treatment were monitored separately for 10 days. Daily records of the number of eggs laid per female, egg clusters per female, and hatched eggs were maintained.

### 4.6. Phylogenetic and Domain Organization Analysis of nAchR

Amino acid sequences of the fruit fly nAchR (Table S1) were obtained from the UniProt server (https://www.uniprot.org/, accessed on 20 July 2023) and used as queries in a BLAST program search (E-value cut-off: 1 × 10^−6^) against predicted proteins in *H. halys* and *A. chinensis*; all candidates were confirmed using the SMART database. Ambiguous positions were removed from each sequence pair prior to analysis. The amino acid sequences of nAchRs from *H. halys* and *A. chinensis* were imported using MEGA (version 10.2.0), and sequence alignments were carried out using the MUSCLE method [53]. The phylogenetic trees of nAchRs were constructed using the neighbor-joining method. Conserved protein domains were identified using the SMART server (http://smart.embl-heidelberg.de/ (accessed on 10 November 2023)) with default parameters, and the results were confirmed using the TMHMM server. The domain distributions of nAChRs were visualized using TubercuList Bioinformatics Tools (TBtools) (version 1.0). The root mean square deviation (RMSD) between each structure was calculated using VMD (version 1.9.4).

### 4.7. Homology Modeling and Docking

The 3D conformer structure of imidacloprid was downloaded from the PubChem database (CID: 86287518). The 3D structures of *A. chinensis* and *H. halys* nAchRs were obtained via homology modeling using PHYRE2 (ic.ac.uk) [54]. PDB files were downloaded from PHYRE2 for structural alignment. Prior to molecular docking, all water molecules were removed from nAchRs. AutoDock Tools (version 1.5.7) was used to generate the input files and identify the grid center. Imidacloprid was docked into the active site of nAchR using AutoDock Vina, and the superior structure with the lowest docked energy was selected for initial configuration in molecular dynamics simulations. Structural alignments and 3D structural figures were generated using PyMOL (version 1.8).

### 4.8. Statistical Analysis

All data were analyzed using SPSS 25 (IBM Corp., Armonk, NY, USA) and Origin 2022. KD_50_ values were determined using a probit regression model [55]. Two-way repeated-measures analysis of variance (ANOVA), with treatment and time as factors, was used to analyze the weight, CO_2_ release rate, and metabolic rate of *A. chinensis*. The normality of continuous variables was assessed using the Shapiro–Wilk test (*p* > 0.05), and the square root transformation (SQRT) function was applied when appropriate. Mauchly’s sphericity test was used to verify sphericity, with adjustments made using the Greenhouse–Geisser (ε < 0.75) or Huynh–Feldt (ε ≥ 0.75) corrections. One-way ANOVA and the Waller–Duncan test were used for other data comparisons. Data are expressed as the mean ± standard error of the mean (SEM). Significance was set at *p* < 0.05.

## 5. Conclusions

Imidacloprid is more harmful to the phytophagous *H. halys* but impairs the physiological activity and reproduction of the predaceous *A. chinensis*. Thus, this pesticide affects the behavior and survival of non-target *A. chinensis* to differing degrees depending on the field dose used. Nonetheless, further research is required to determine the precise mechanism by which the neonicotinoid affects non-target insects.

## Data Availability

The original contributions presented in this study are included in the article/Supplementary Material. Further inquiries can be directed to the corresponding author.

## Supplementary Materials

The following supporting information can be downloaded at: https://www.mdpi.com/article/10.3390/ijms26020690/s1.
