# Supplementary material for: Predaceous and Phytophagous Pentatomidae Insects Exhibit Contrasting Susceptibilities to Imidacloprid
Source: Int J Mol Sci. 2025 Jan 15;26(2):690. doi: 10.3390/ijms26020690 (PMC11766023; doi:10.3390/ijms26020690)
Supplement: Supplementary file 1 [file ijms-26-00690-s001.zip › ijms-3340887-supplementary.pdf]

# Supplementary Materials for:

Predaceous and Phytophagous Pentatomidae Insects Exhibit Contrasting Susceptibilities to Imidacloprid

**Table S1 Query sequences of the published *Drosophila melanogaster* proteins**

| Entry | Entry Name | Protein Name | Gene Name | Organism | Length (AA) |
|---|---|---|---|---|---|
| P04755 | ACH3_DROME | Acetylcholine receptor subunit beta-like 1 | nAChRbeta1, Acr64B, AcrD, ard, nAcRbeta-64B, CG11348 | *Drosophila melanogaster* (Fruit fly) | 521 |
| P17644 | ACH2_DROME | Acetylcholine receptor subunit alpha-like 2 | Acetylcholine receptor subunit beta-like 2 | *Drosophila melanogaster* (Fruit fly) | 576 |
| P09478 | ACH1_DROME | Acetylcholine receptor subunit alpha-like 1 | Acetylcholine receptor subunit beta-like 2 | *Drosophila melanogaster* (Fruit fly) | 567 |
| P25162 | ACH4_DROME | Acetylcholine receptor subunit beta-like 2 | nAChRbeta2, Acr96Ac, AcrF, nAcRbeta-96, SBD, CG6798 | *Drosophila melanogaster* (Fruit fly) | 519 |

**Table S2 nAchR subunit protein ID of *Arma chinensis* and *Halymoropha halys* (NCBI)**

| Protein ID | Name in phylogenetic tree |
|---|---|
| evm.model.contig1305.34 | AchR beta-like1 [ARMA] |
| evm.model.contig16.19 | AchR beta-like2 [ARMA] |
| evm.model.contig16.16 | AchR alpha [ARMA] |
| evm.model.contig265.1 | AchR alpha3-like1 [ARMA] |
| evm.model.contig1093.7 | AchR alpha-like2 [ARMA] |
| evm.model.contig1114.9 | AchR non-alpha3-like [ARMA] |
| evm.model.contig374.87 | AchR alpha3-like [ARMA] |
| evm.model.contig292.10 | AchR alpha5-like [ARMA] |
| XP_024215583.1 | AchR beta-like1 [BMSB] |
| XP_024214307.1 | AchR alpha-like X2 [BMSB] |
| XP_024214306.1 | AchR alpha-like X1-2 [BMSB] |
| XP_014287844.1 | AchR alpha-L1 [BMSB] |
| XP_014289315.1 | AchR alpha-like X1-1 [BMSB] |
| XP_014289314.1 | AchR alpha-like X1 [BMSB] |
| XP_014289316.1 | AchR alpha-like X2-1 [BMSB] |
| XP_014289941.2 | AchR alpha-like1 [BMSB] |
| XP_014284142.1 | AchR alpha7 X1 [BMSB] |
| XP_014284143.1 | AchR alpha7 X2 [BMSB] |
| XP_014289317.1 | AchR alpha-like X3 [BMSB] |
| XP_014282440.2 | AchR alpha7-like X1 [BMSB] |
| XP_014282438.2 | AchR alpha7-like X4 [BMSB] |
| XP_014291973.1 | AchR beta-like2 [BMSB] |
| XP_014282442.2 | AchR alpha7-like X2 [BMSB] |
| XP_014284145.1 | AchR alpha7 X4 [BMSB] |
| XP_014282443.2 | AchR alpha7-like X3-1 [BMSB] |
| XP_014284144.1 | AchR alpha7 X3 [BMSB] |
| XP_014292243.1 | AchR alpha3-like [BMSB] |

| | |
|---|---|
| XP_014287843.1 | AchR beta-like2-1 [BMSB] |
| XP_014280625.1 | AchR alpha-like2 [BMSB] |
| XP_014273508.1 | AchR non-alpha3-like [BMSB] |
| XP_014280467.1 | AchR beta3-like [BMSB] |
| XP_014277544.1 | AchR alpha5-like [BMSB] |

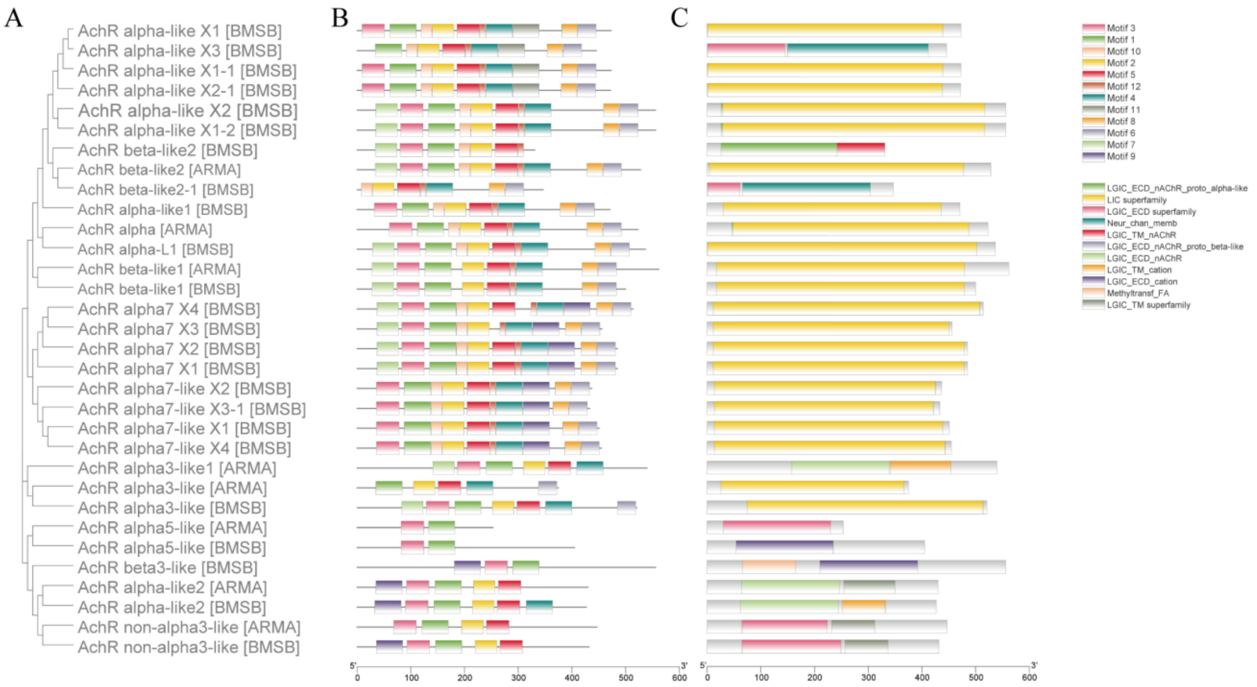


**Figure S1.** Phylogenetic relationships, protein motif analyses, and domain analysis of *Arma chinensis* and *Halyomorpha halys*. (A) Phylogenetic tree of *A. chinensis* and *H. halys* AchR. (B) Nicotinic acetylcholine receptor (nAchR) motifs identified using MEME (Version 5.5.3). (C) Domain organization of nAchRs, with different colors and shapes representing the domains and regions. Graphics drawn with TBtools.

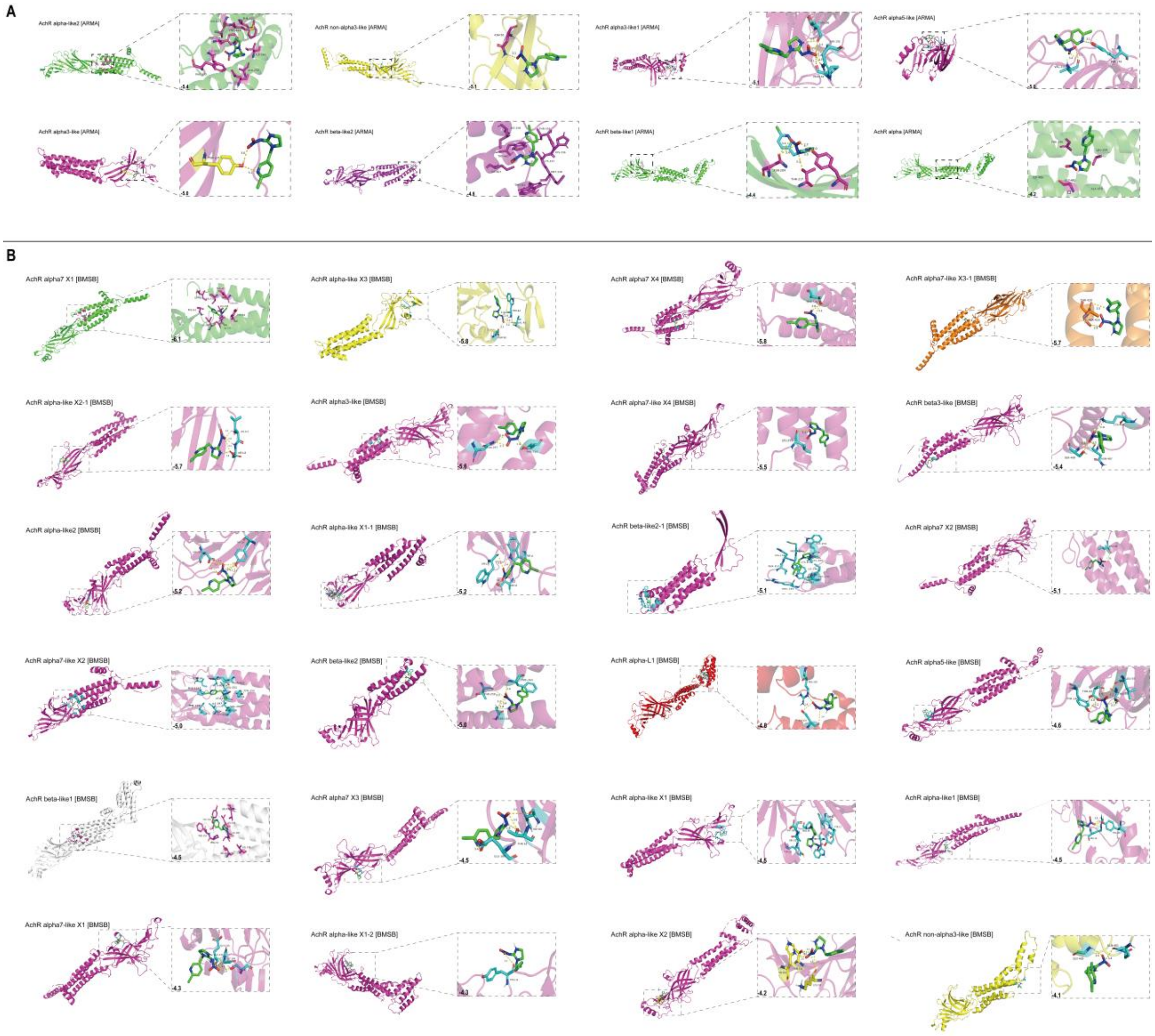


**Figure S2.** Molecular docking of imidacloprid with acetylcholine receptor subunits *Arma chinensis* and *Halyomorpha halys*. Binding mode of the nAChR of *A. chinensis* (A) and *H. halys* (B) to imidacloprid, with wide-angle view (left) and focused image (right). Docking represents one of the five subunits comprising the nAChR, showing imidacloprid binding at < 4.0 Å.
